# The Magnitude of Tobacco Smoking-Betel Quid Chewing-Alcohol Drinking Interaction Effect on Oral Cancer in South-East Asia. A Meta-Analysis of Observational Studies

**DOI:** 10.1371/journal.pone.0078999

**Published:** 2013-11-18

**Authors:** Stefano Petti, Mohd Masood, Crispian Scully

**Affiliations:** 1 Department of Public Health and Infectious Diseases, Sapienza University, Rome, Italy; 2 Centre of Studies for Community Dentistry, Faculty of Dentistry, Universiti Teknologi Mara, Shah Alam, Malaysia; 3 University College London, London, United Kingdom; MOE Key Laboratory of Environment and Health, School of Public Health, Tongji Medical College, Huazhong University of Science and Technology, China

## Abstract

Tobacco smoking, betel quid chewing and alcohol drinking are oral cancer risk factors. Observational studies unanimously report that oral cancer risk in smoking-drinking-chewing exposed subjects is exceptionally high. However, none of them assessed the fractions of this risk attributable to the three individual risk factors and to the smoking-drinking-chewing interaction. The present study sought to assess the magnitude of the smoking-drinking-chewing interaction effect on oral cancer. A meta-analysis of observational South-East Asian studies which reported oral cancer odds ratios (ORs) stratified for smoking-drinking-chewing exposures was performed. The pooled ORs were estimated and controlled for quality, heterogeneity, publication bias and inclusion criteria. The smoking-drinking-chewing interaction effect was estimated through the pooled Relative Excess Risk due to Interaction (RERI, excess risk in smoking-drinking-chewing exposed individuals with respect to the risk expected from the addition of the three individual risks of smoking, drinking and chewing). Fourteen studies were included with low between-study heterogeneity. The pooled ORs for smoking, drinking, chewing, smoking-drinking-chewing, respectively were 3.6 (95% confidence interval −95% CI, 1.9–7.0), 2.2 (95% CI, 1.6–3.0), 7.9 (95% CI, 6.7–9.3), 40.1 (95% CI, 35.1–45.8). The pooled RERI was 28.4 (95% CI, 22.9–33.7). Among smoking-drinking-chewing subjects, the individual effects accounted for 6.7% (smoking), 3.1% (drinking), 17.7% (chewing) of the risk, while the interaction effect accounted for the remaining 72.6%. These data suggest that 44,200 oral cancer cases in South-East Asia annually occur among smoking-drinking-chewing exposed subjects and 40,400 of these are exclusively associated with the interaction effect. Effective oral cancer control policies must consider concurrent tobacco smoking, alcohol drinking, betel quid chewing usages as a unique unhealthy lifestyle.

## Introduction

In South-East Asia, oral cancer is the second most frequent form of cancer and the second most frequent cause of death from cancer among males. One third of global cases and one half of deaths from oral cancer occur in this region [1]. These high incidence and mortality rates are due to lifestyle risk factors such as tobacco smoking, betel quid chewing and alcohol drinking [2]–[5], which are frequent in this region, as well as to genetic and infectious factors [6]–[8]. Tobacco use is widespread in South-East Asia and male smoking rate is recorded close to 50% in most countries, but the actual tobacco smoking rate is probably higher, due to cigarette smuggling and to various unrecorded forms of tobacco consumption modalities, such as bidi, kreteks, sulpa, chilum, hookli and waterpipes, which may account for more than one half of the total amount of smoked tobacco [9], [10]. Betel quid/areca nut chewing is widespread with chewing rates as high as 30–40% among adults. There is a great spectrum of ingredients and patterns of consumption. For example, areca nut is prepared as green unripe, fermented, boiled, sweetened, while betel leaves and/or inflorescence can be used. In addition, there can be various other ingredients, such as tobacco, spices, sweeteners, lime and catechu [11], [12]. Alcohol drinking is also widespread in South-East Asia and drinking rates are higher than the rates reported by the national statistics [13], because of unrecorded alcoholic beverage production, which includes home brewing, illicit production, alcohol imported illegally and smuggling. Local products, such as arrack, toddy, oou, bangla mad are regularly consumed by adults and even adolescents, mostly males, and adult drinking rates as high as 50% are reported [14], [15].

Oral cancer patients from South-East Asia are, therefore, frequently exposed to one or more of these lifestyle risk factors [16] and, unsurprisingly, oral cancer risk is extremely high in smoking-drinking-betel quid chewing individuals, as noted by Notani in 1988, who reported that in multi-exposed individuals oral cancer risk was fifty times higher than in unexposed individuals [17]. Many observational studies have confirmed this first observation (reviewed by IARC in [3], [18]).

The oral cancer risk in individuals exposed to smoking, drinking and betel quid chewing is often higher than the sum of the individual risks of smoking, drinking and betel quid chewing. Such an additional risk due to concurrent exposure is termed the interaction or joint effect. An example of an interaction effect on oral cancer is the concurrent exposure to smoking and drinking. According to a large case-control study from Brazil, the first which made an adjustment for confounding and for interaction, three quarters of the overall oral cancer risk in multi-exposed individuals was due to such a joint effect and only one quarter was due to the sum of the independent effects of smoking and drinking [19]. Two multi-centre studies, namely, the International Head and Neck Cancer Epidemiology (INHANCE) and the Alcohol-Related Cancers and Genetic susceptibility in Europe (ARCAGE) reported that the smoking-drinking interaction was per se responsible for 40% of oral cancer cases [20], [21]. Finally, a meta-analysis of observational studies estimated that the interaction effect was responsible for more than one half of the overall cases of oral cancer [22].

The hypothetical interaction effect of smoking, drinking and betel quid chewing on oral cancer has never been estimated, however. Therefore, the aim of the present meta-analysis of observational studies was to explore and assess the interaction effect of tobacco smoking, alcohol drinking and betel quid chewing on oral cancer risk in South-East Asian countries, where concurrent exposure to these risk factors is widespread.

## Methods

A literature search, limited to the year range 1988–2013, was made by the three authors independently. The matched terms used were: (1) Oral cancer, mouth cancer, head and neck cancer, upper aero-digestive tract cancer; (2) Betel, areca, paan masala, gutkha, chew*, chewing; (3) Alcohol, drinking, drink*, alcoholic beverage, ethanol; (4) Tobacco, cigarette, bidi, smoke, smok*, smoking.

Databases used were Medline, through PubMed (C.S.) and Ovid (M.M.), and Scopus (S.P.). Other studies were located using the reference lists of identified studies and Google Scholar.

Eligible observational studies showed the following characteristics: (1) Subjects were adults from South-East Asia. Studies on immigrants from these countries to Western countries were not considered however, since subjects could have changed their lifestyle in their new context; (2) Case patients were affected by squamous cell carcinoma of mouth and/or oro-pharynx (International Statistical Classification of Diseases and Related Health Problems, 10^th^ version, ICD-10, codes C00–C06, C09, C10) confirmed clinically and histologically. Studies which made no discrimination between oral/oro-pharyngeal cancer and cancers of major salivary glands, pharynx, oesophagus and larynx were not considered; (3) Control patients could be affected by control diseases, but were not affected by other forms of cancer or oral potentially malignant disorders, such as erythroplakia or leukoplakia. Controls could be selected either from the same hospitals where cases were selected or from the underlying study populations. Studies which used population-based controls extracted from other studies were not considered, as they could be subjected to information bias due to different methods used to assess patients' exposures [23]; (4) Exposures were assessed using history/anamnesis/questionnaire at the time of diagnosis. Exposed subjects were daily users for at least five years whatever the level of consumption. Occasional users, former users, or daily users exposed for less than five years were not considered for the meta-analysis.

This study search provided a large number of studies, few relevant for the purpose of the present analysis. Therefore, a preliminary list of potential primary studies was made on the basis of the information gathered from titles and abstracts. Full texts of the remaining studies were obtained and those with the aforementioned characteristics which, in addition, provided the numbers of cases and controls stratified for all the various smoking-drinking-betel quid chewing exposure categories, were selected. These categories were, non-smoking/non-drinking/betel quid non-chewing subjects (unexposed), smoking/non-drinking/betel quid non-chewing subjects (SM), non-smoking/drinking/betel quid non-chewing subjects (DR), non-smoking/non-drinking/betel quid chewing subjects (BQ), smoking/drinking/betel quid non-chewing subjects (SM/DR), smoking/non-drinking/betel quid chewing subjects (SM/BQ), non-smoking/drinking/betel quid chewing subjects (DR/BQ), and smoking/drinking/betel quid chewing subjects (SM/DR/BQ). Corresponding authors of studies which met the inclusion criteria but did not provide the numbers of cases and controls stratified for all the various SM/DR/BQ exposure categories were contacted via email to obtain these data. After this process, the list of primary studies to include in the present meta-analysis was set through discussions and approved by all authors.

Data were extracted by the three reviewers independently, the results were compared and the differences reconciled through discussions. The oral cancer odds ratios (ORs) with 95% confidence intervals (95% CIs) for each exposure category were assessed.

Primary study quality was rated by the three reviewers on the basis of the study design (e.g., adequateness, consistency of diagnoses, etc.), giving score 1.0 to high-quality studies, 0.5 to moderate-quality studies, 0.25 to low-quality studies. It must be anticipated that all the studies used for the present analysis were given score 0.5, therefore, this quality score was not applied because it did not change the pooled risk estimates [24].

Exposures were treated dichotomously, that is, ever (routine) usage vs. never, excluding occasional and former usage. Such an exposure categorization increased the reliability of the pooled risk estimates, although it did not consent to make any distinction between various forms of exposure, such as type of product, pattern of consumption, etc. [25], [26].

Publication bias was explored for each exposure category separately because it was assumed that the degree of this form of bias could be different among the different SM/DR/BQ exposure categories. Indeed, some of these categories included only a few subjects and, consequently, oral cancer ORs in these categories were less reliable than the risk estimates in the remaining exposure categories. A visual preliminary investigation was made using the funnel plots, with the ln(OR) in the *x*-axis and precision, that is, 1/[standard error ln(OR)] in the *y*-axis. An asymmetrical plot was suggestive of high level of publication bias. Formal correction for publication bias was made including in the set of primary studies one or more missing studies, which were identified using the R_0_ method. The funnel plot was drawn after the inclusion of missing studies and compared with the plot drawn without missing studies to see whether symmetry was improved [27]–[29].

The pooled oral cancer ORs (pORs) were estimated for every exposure category. The method used for the assessment was chosen on the basis of the level of between-study heterogeneity. Heterogeneity was estimated with the Cochran's Q, a χ^2^ test with (k-1) degrees of freedom, where k is the number of primary studies. For Q≤(k-1) the level of heterogeneity was low enough and the fixed-effects method was used, with the inverse of the variance of ln(OR) as study weight. For Q>(k-1) the level of heterogeneity was high and required the use of the more conservative random-effects method [24].

Sensitivity analysis to study inclusion was performed [30] to investigate whether the pooled OR estimates were excessively influenced by a single study. For every exposure category, the contribution of each study to the overall weight was measured as a percent of the total weight. Studies which yielded weights ≥20% were likely to exert a great influence on the pooled risk estimates and therefore were excluded in turn. The pOR was re-estimated and compared with the overall pOR. If the 95% CIs of the two pORs did not overlap, the pooled risk estimate for that exposure category was regarded as not robust enough [24].

The fundamental aim of the present meta-analysis was to investigate the SM/DR/BQ interaction effect on oral cancer. Therefore, if the pOR in this multi-exposure category was larger than the sum of the pORs of SM, DR, BQ, interaction on an additive scale, also known as departure from additivity, was present. Interaction on a multiplicative scale, or departure from multiplicativity, could occur if the SM/DR/BQ pOR was larger than the pORs for SM, DR and BQ multiplied by each other. Departure from additivity does not exclude departure from multiplicativity, departure from multiplicativity includes departure from additivity, the lack of departure from multiplicativity does not preclude departure from additivity [31]. Therefore, in order to ascertain whether an interaction effect of any kind was present, it was assessed on additive scale.

The assessment of the interaction effect using risk estimates, such as OR or Relative Risk (RR), is based on the concept of Relative Excess Risk (RER), which is the excess risk in individuals exposed to a given risk factor with respect to the risk in unexposed individuals (therefore, RER_unexposed_ = 0), with formula:
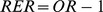
Therefore, in case of exact additivity and no interaction: 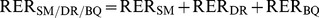
Or, substituting (OR – 1) to RER:
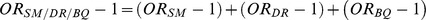
and then,
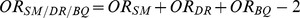
If there was departure from additivity and interaction effect, the RER_SM/DR/BQ_ was greater than the sum of the of the individual RERs [32]:
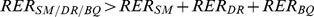
and then, remembering “RER = OR – 1”,
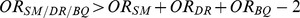
The difference between the two sides of the equation is known as Relative Excess Risk due to Interaction (RERI) and can be interpreted as the excess risk in SM/DR/BQ exposed individuals with respect to the risk that is expected from the sum of the three individual risks. The RERI formula is, therefore:
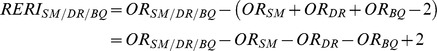
Thus, if RERI_SM/DR/BQ_ = 0 there was exact additivity and no interaction, if RERI_SM/DR/BQ_>0, there was interaction.

Although RERI is not the only way to estimate the magnitude of the interaction effect (there are other measures, such as the Attributable Proportion due to Interaction –API, and the Synergy Index -S), it was chosen, as it was considered the most intelligible and reliable method. Indeed, the use of attributable proportions in multifactorial models, often leads to a sum of proportions higher than 100%, which may sound astonishing and difficult to interpret for readers who are not expert in epidemiology [33], while S is generally statistically more unstable than RERI and API, when it is estimated using ORs instead of RRs [34].

One major problem with RERI and other formal measures of interaction is the assessment of the confidence interval. Whenever RERI>0 there is evidence for interaction in the sample under investigation, thus making CI assessment unnecessary, but if the analysis seeks to make a RERI estimate which could be extended outside the confines of the study, 95% CI assessment becomes mandatory. There are several possibilities for calculating 95% CI [35], the method with the best performance in simulation studies, that does not require logistic regression analysis –and is, therefore, applicable to meta-analyses, is based on two-by-four tables for two risk factors [36], and, for extension, two-by-five tables for three risk factors. The good points of this formula to estimate the 95% CI of RERI_SM/DR/BQ_ are that it is computable using hand-held calculators and, very importantly, it accounts for the pairwise correlations between ORs. In fact, OR_SM/DR/BQ_ is necessarily correlated with OR_SM_, OR_DR_, OR_BQ_ and these individual ORs are necessarily inter-correlated, thus, CI assessment without accounting for these correlations leads to inflated and often unreliable CI estimates. Correlation coefficients were estimated through the variances of the ln(pOR)s and the numbers of unexposed cases and unexposed controls, obtained from the sum of these figures reported by the primary studies. The formula with three risk variables used for the present analysis was derived from the original formula with two variables reported by Zou [36]. Using the same method, RERI_SM/DR_, RERI_SM/BQ_ and RERI_DR/BQ_ were estimated, to assess the interaction effects of the investigated risk factors in the SM/DR, SM/BQ and DR/BQ categories.

Subgroup analysis was planned and was considered a type of sensitivity analysis. The differences between studies according to age, gender and country, as surrogate marker of ethnicity, were assessed informally, since authors generally adopted different distribution criteria (e.g., means, frequency distributions, etc.). If evident between-study differences emerged, subgroup analysis was performed: studies were stratified according to age, gender or country and RERI_SM/DR/BQ_ in the various subgroups were assessed and compared. The covariates used by each primary study to adjust the OR estimates also were listed and, in the event that studies were largely different according to their number and type, subgroup analysis was performed and studies were stratified for number/type of covariates used.

The proportion of oral cancer cases which annually occur in South-East Asia exclusively due to the SM/DR/BQ interaction was approximately estimated. The formula for the assessment of the Population Attributable Risk Fraction (PAF), that is,
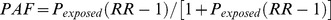
was used. Prevalence of SM/DR/BQ exposed individuals in the general adult population was estimated as weighted mean of data on exposure from literature using the inverse of variance as weight. The overall proportion of cases which occurred among SM/DR/BQ exposed subjects was preliminarily assessed substituting “RR – 1” with “OR_SM/DR/BQ_ – 1”. The proportion of cases exclusively attributable to the SM/DR/BQ interaction was assessed substituting “RR – 1” with RERI_SM/DR/BQ_.

The statistical software StatView 5.0.1 (SAS® Institute Inc., NC, USA) was used for the statistical analyses. The level of significance was set at 95%.

This paper follows the MOOSE guidelines for reporting meta-analyses of observational studies [37].

## Results

Eighty-four studies were considered potentially eligible for inclusion, on the basis of titles and abstracts. Forty-seven of these were then excluded because the case definition did not fall within the present inclusion criteria or exposures to smoking, drinking and betel quid chewing were not assessed. Of the remaining studies, twenty-two were excluded: in eighteen of them, which focused on genetic factors, lifestyle variables were used for OR adjustments, while in four other studies stratified data were not reported and corresponding authors failed to provide them. Thus, fourteen studies remained and were used for the meta-analysis (flow chart in **Appendix S1**, list in **Table 1**) [38]–[51]. The ORs for all the exposure categories were assessed using the crude data and are shown in **Appendix S2**. The point estimates for oral cancer ORs in the SM/DR/BQ category ranged between 4.6 (study 10) and 80.4 (study 2) and were the highest among all the various exposure categories, excluding study 5, where OR_SM/BQ_ was slightly higher (48.6 OR_SM/DR/BQ_ vs. 48.8 OR_SM/BQ_).

**Table 1:** General characteristics of the primary studies.

First author, year	Number	Country, year of the survey	Mean age	Gender (% males)	Overall cases	Overall controls
Chang, 2011	1	Taiwan, 2005–2010	54,6	100%	285	13,321
Lee, 2012	2	Taiwan, 2000–2007	54,8	87.2%	810	2250
Lin, 2011	3	Taiwan, 2005–2008	55.2	100%	230	10,257
Lohe, 2010	4	India, 2009	42.0	66.2%	70	70
Tsai, 2009	5	Taiwan, 2003–2005	55.0^a^	100%	239	1,370
Yen, 2008	6	Taiwan, 2005–2007	55,3	100%	191	8,080
Subapriya, 2007	7	India, 1991–2003	47.4	52.1%	388	378
Yang, 2007	8	Taiwan, 2005–2006	55.8	100%	131	5,640
Ko, 1995	9	Taiwan, 1992–1993	48.0	97.2%	107	200
Muwonge, 2008	10	India, 1996–2004	59.0^a^	57.8%	163	815
Znaor, 2003	11	India, 1993–1999	50.5^a^	100%	1,377	3,634
Sankaranarayanan, 1989	12	India, 1983–1984	59.0^a^	60.5%	83	501
Sankaranarayanan, 1990	13	India, 1983–1984	57.2^a^	60.8%	414	895
Rao, 1994	14	India, 1980–1984	47.8^a^	100%	704	630

a estimated from the frequency distribution.

Seven primary studies had been conducted in India and a further seven in Taiwan (**Table 1**), this balanced distribution suggested that subgroup analysis stratified for country, surrogate marker of ethnicity, was mandatory. The mean ages ranged between 42 (study 4) to 59 years (studies 10 and 12). Males were always largely prevailing, ranging between almost 60% (study 10) and 100% (studies 1, 3, 5, 6, 8, 11, 14). These similar age and gender distributions suggested that age/gender-based subgroup analyses were unnecessary. Covariate-based subgroup analysis also was unnecessary, because some primary studies did not report the covariates used to adjust the ORs (studies 1, 3, 6, 8), while for the remaining studies the crude ORs were used.

Some exposure categories, such as SM and SM/BQ, showed symmetrical funnel plots and suggested that publication bias level was low (**Appendix S3**). Conversely, funnel plots for other categories, such as BQ and SM/DR/BQ, were clearly asymmetrical. According to the R_0_ method, the BQ, SM/BQ and SM/DR/BQ categories required an adjustment. More specifically, there were two missing studies, counterparts of studies 5 and 9, for the BQ category; two missing studies, counterparts of studies 4 and 7, for the SM/BQ category; three missing studies, counterparts of studies 4, 10, 14, for the SM/DR/BQ category (data not in Table). The resulting funnel plots, completed with missing studies, were symmetrical (**Appendix S3**).

The Cochran's Q values were low in all exposure categories, excluding SM (**Appendix S4**), which, therefore, was the only category with high level of between-study heterogeneity that required the random-effect method to estimate the pORs. In the remaining exposure categories the fixed-effects method was used. The individual oral cancer pORs were 3.6 (95% CI, 1.9–7.0), 2.2 (95% CI, 1.6–3.0) and 7.9 (95% CI, 6.7–9.3) for SM, DR and BQ, respectively (**Table 2**). The pOR_DR/BQ_ and pOR_SM/BQ_ were higher than the pOR_SM/DR_. The pOR_SM/DR/BQ_ was considerably higher than the other risk estimates (pOR, 40.1; 95% CI, 35.1–45.8).

**Table 2:** Pooled ORs and 95% confidence intervals (95% CIs) for oral cancer adjusted for publication bias in the various exposure categories.

Smoking	Drinking	Chewing	Pooled OR	95% CI
YES	NO	NO	3.63	1.94–7.04
NO	YES	NO	2.20	1.62–2.98
NO	NO	YES	7.90	6.71–9.30
YES	YES	NO	6.29	5.41–7.32
YES	NO	YES	16.01	13.67–18.75
NO	YES	YES	10.44	8.02–13.60
YES	YES	YES	40.09	35.06–45.83

The analysis of study weights revealed that there were one or two studies for each exposure category having relative weights higher than 20%, excluding SM category where all relative weights were lower than 10%, due to the random-effects method (**Appendix S5**). Sensitivity analysis, performed excluding these studies, produced pOR estimates which partly overlapped the pORs estimated without study exclusion and, therefore, corroborated the robustness of risk estimates (**Appendix S6**).

**Figure 1:**
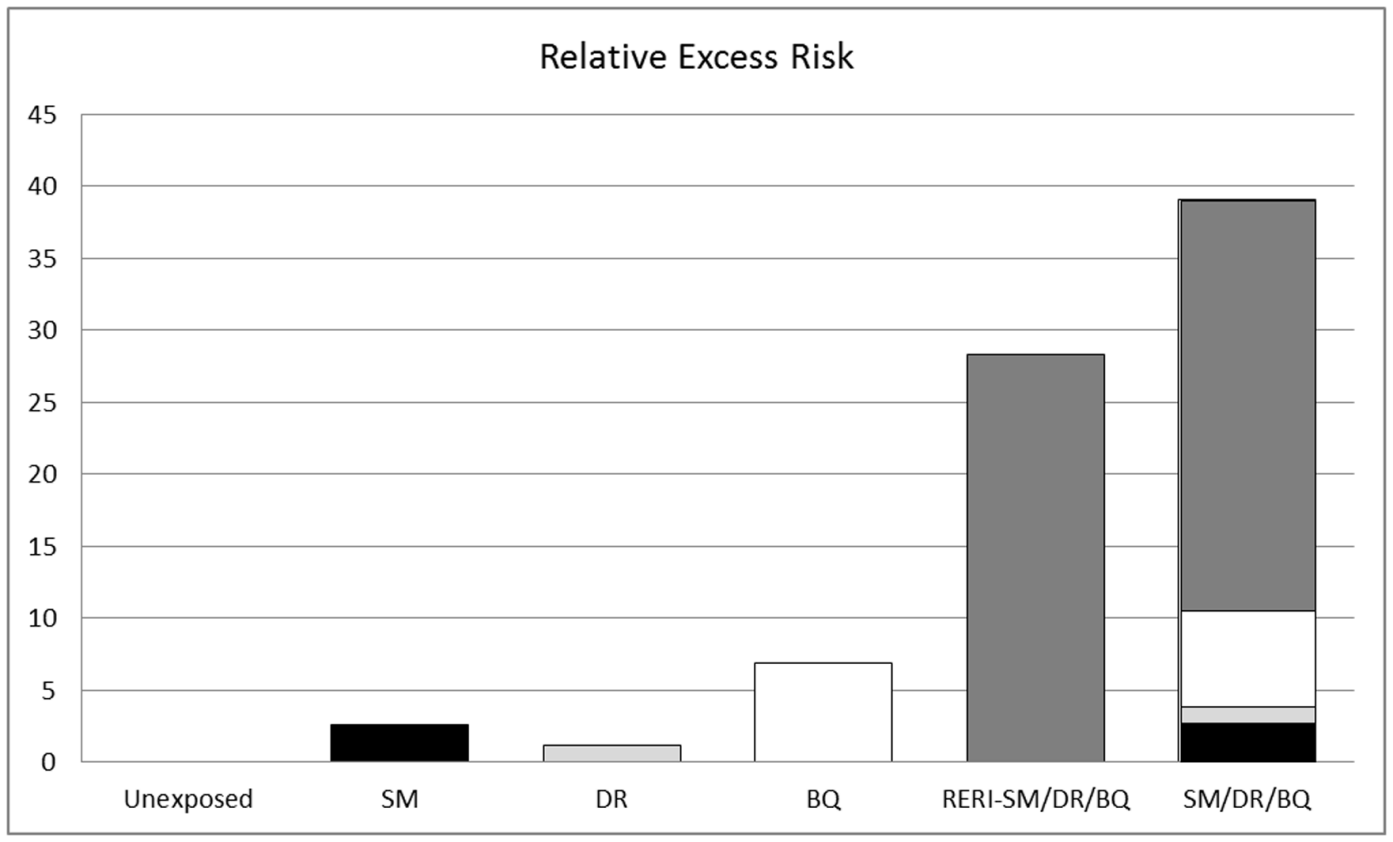
Components of the Relative Excess Risk (RER) in the category of SM/DR/BQ. In unexposed subjects there was no RER (RER_unexposed_ = 0), as these subjects were the reference group. RER_SM_ (in black) accounted for 6.7% of RER_SM/DR/BQ_. RER_DR_ (in light grey) accounted for 3.1% of RER_SM/DR/BQ_. RER_BQ_ (in white) accounted for 17.7% of RER_SM/DR/BQ_. The SM/DR/BQ interaction effect, that is, the Relative Excess Risk due to Interaction (RERI) between SM, DR and BQ (RERI_SM/DR/BQ_, in dark grey) accounted for 72.6% of RER_SM/DR/BQ_.

The pooled RERI_SM/DR/BQ_ was 28.4 (95% CI, 22.9–33.7) and was considerably higher than the pooled RERI_SM/DR_, RERI_BR/BQ_ and RERI_SM/BQ_ which were not significant or marginally significant (**Table 3**). **Figure 1** shows the components of the Relative Excess Risk (RER) in the SM/DR/BQ exposure category. RER for unexposed subjects, the reference group, was zero. The individual effects of SM, DR and BQ accounted for 6.7%, 3.1% and 17.7% of the overall RER_SM/DR/BQ_, respectively. The pooled SM/DR/BQ joint effect, that is, the pooled RERI_SM/DR/BQ_, accounted for 72.6% of RER_SM/DR/BQ_, almost three quarters of the excess risk in this multi-exposure category.

**Table 3:** Pooled interaction effects of smoking, drinking and betel quid chewing assessed through the Relative Excess Risk due to Interaction (RERI).

Joint exposure categories	Pooled RERI	95% CI
Smoking-Drinking	1.46	−2.06–3.40
Smoking-Betel Quid Chewing^a^	5.48	1.06–8.20
Drinking-Betel Quid Chewing	1.34	−1.29–4.50
Smoking-Drinking-Betel Quid Chewing^a^	28.36	22.92–33.74

a p<0.05.

RERI higher than 0 denoted a significant joint effect.

The subgroup analysis with the primary study set stratified into Indian and Taiwanese studies is shown in **Table 4**. The pooled oral cancer OR estimates were higher in the Taiwanese studies than in the Indian studies in the three exposure categories of BQ, SM/BQ and DR/BQ. However, the pOR_SM/DR/BQ_ was similar in both study groups (Indian studies, pOR 46.1, 95% CI, 38.1–55.7; Taiwanese studies, pOR 55.1, 95% CI, 37.0–82.3). These data provided RERI_SM/DR/BQ_ estimates of 38.1 and 36.4 for Indian and Taiwanese studies respectively, thus corroborating the reliability of the estimates of this meta-analysis. The estimated pooled SM/DR/BQ interaction effects accounted for 84.6% and 67.3% of the RER_SM/DR/BQ_ in India and Taiwan, respectively.

**Table 4:** Pooled ORs for oral cancer in the various exposure categories and pooled RERI for smoking, drinking and betel quid chewing (95% CIs between brackets).

Pooled estimate	Indian studies	Taiwanese studies
OR_SM_	2.92 (2.44–3.49)	3.90 (3.06–4.95)
OR_DR_	2.69 (1.73–4.18)	1.80 (1.17–2.77)
OR_BQ_ a	7.03 (5.87–8.41)	15.03 (9.87–22.87)
OR_SM/DR_	5.81 (4.81–7.03)	7.17 (5.64–9.12)
OR_SM/BQ_ a	9.87 (8.08–12.06)	35.87 (27.66–46.53)
OR_DR/BQ_ a	5.05 (3.63–7.03)	36.23 (23.57–55.70)
OR_SM/DR/BQ_	46.06 (38.09–55.70)	55.14 (36.97–82.27)
RERI_SM/DR/BQ_ b	38.11 (30.05–41.62)	36.42 (24.87–53.68)

a p<0.05.

b Percent of overall RER_SM/DR/BQ_ accounted by RERI_SM/DR/BQ_: 84.6% (Indian studies), 67.3% (Taiwanese studies).

Subgroup analysis with primary study set stratified into Indian (studies, 4, 7, 10–14) and Taiwanese (studies, 1–3, 5, 6, 8, 9) studies.

The prevalence estimates of SM/DR/BQ exposed individuals in South-East Asia reported by the most recent literature data were 6.59% (95% CI, 5.85–7.33%) [52] and 9.00% (95% CI, 8.16–9.84%) [53]. The resulting weighted mean was 7.64%. Therefore, the proportion of oral cancer cases which annually occur in South-East Asia and are attributable to concurrent SM/DR/BQ exposure is 74.92%. The proportion exclusively attributable to the SM/DR/BQ interaction was 68.42% (data not in Table).

## Discussion

This study endeavoured to avoid the publication bias so frequent in meta-analyses of observational studies and typical of papers which do not find significant associations between risk factors and outcome [24]. In order to achieve this, two methods were used to control for publication bias and to detect potentially missing studies. The fact that BQ, SM/BQ and SM/DR/BQ resulted in the three exposure categories with a high degree of publication bias supported the adequateness of this protocol. Indeed, these exposures are those most typically seen in South-East Asia. In Taiwan, for example, 17% adults chew betel quid, 14% smoke cigarettes and chew betel quid and 9% smoke cigarettes, chew betel quid and drink alcoholic beverages [53]. It is likely that any papers which did not find significant associations between these typical behaviours and oral cancer were never published or, if they were published, non-significant associations were not considered interesting and were not shown.

The present meta-analysis was potentially subject however, to the forms of bias frequent in case-control studies, i.e., information, recall, interviewer and selection bias. Information bias is typical in studies which assess exposures from the history. Indeed, heavy users may under-report their exposure level, while other individuals may change their lifestyle in the course of their life, by increasing the consumption level progressively, starting joint consumptions, or changing the types of products used, or consumption frequency and modality etc. [23]. Therefore, information regarding exposure is notoriously unreliable when classified quantitatively according to consumption frequency and years of usage, or qualitatively according to type of products used [54]–[56]. In order to endeavour to control for information bias, exposures to SM, DR, BQ were therefore classified into broad categories, namely, ever (routine) vs. never usage, excluding former and occasional usage. This choice provides less specific but more reliable information but was preferred to the alternative of providing more analytical, but less consistent information -an approach generally preferred by experts in the epidemiology of lifestyle risk factors [26], [57]. Recall bias may have a negative impact on case-control studies due to systematic differences between cases and controls in reporting exposures, because some oral cancer patients may have pondered on the lifestyle that might have caused their condition, thus over-reporting their exposures [23], but such an assumption is not justified in the present context, because the majority of the adult male population has a low level of awareness toward behavioural oral cancer risk factors [58]–[60]. In order to control for selection bias, a prerequisite for eligibility of primary studies was that the authors had selected population-based controls (as in studies 5 and 10), or hospital-based controls with subjects who were not affected by oral precancerous lesions, other diseases promoted by the risk factors under investigation, or other cancers (as in the remaining studies included) [23].

Another potential limitation of this meta-analysis is that different studies may have accounted for different sets of covariates, thus making the various OR estimates incomparable. Oral cancer aetiology is multifactorial and many behavioural, genetic, environmental factors, concur in its development and progression [61], [62] and there may even be unknown factors. Thus, a meta-analysis of observational studies which accounts for all the possible covariates is probably unfeasible. Subgroup analysis was designed to account for between-study differences with respect to age/gender distribution, ethnicity and covariates used in the multivariate analysis. However, such an analysis was limited to the only ethnicity and the pooled SM-DR-BQ joint effects in Indian studies and in Taiwanese studies were almost totally overlapping (**Table 4**). In addition, the analysis of between-study heterogeneity showed that the primary studies resulted homogeneous (**Appendix S4**), an uncommon situation in meta-analyses of observational studies [24], probably because studies were performed in the same area. An important consequence of this is that, in homogeneous samples, the hidden, non-investigated and unknown factors are considered part of the background environment, assumed to be uniformly distributed and can be disregarded [23], [32].

Despite these limitations, the merit of the present meta-analysis was to be the first to provide a formal and reliable estimate of the interaction effect of concurrent SM, DR, BQ on oral cancer. Many observational studies from South-East Asia have emphasized that oral cancer risk in SM/DR/BQ exposed subjects was exceptionally high (reviewed by [3], [18]) and similar results were also reported by studies investigating oral potentially malignant disorders (OPMDs, i.e., leukoplakia, erythroplakia, lichen planus, submucous fibrosis, verrucous lesions) (see, for example, [63]–[64]). Nevertheless, none of these reports investigated how much of the oral cancer burden was due to the SM/DR/BQ interaction, an essential aspect for the design of effective oral cancer control policies in South-East Asia. Indeed, 59,000 of the 170,000 world annual oral cancer cases among males occur in the South-East Asian region [1]. According to the present analysis 44,200 of these cases (74.92% of 59,000, as in the Results section) occur among SM/DR/BQ exposed subjects. The majority of these cases (40,400, that is, 68.42% of 59,000, as in the Results section) are exclusively due to the SM/DR/BQ interaction. Such an interaction effect seems also associable with the development of premalignant lesions. Indeed, using the ORs for the various exposure categories reported by a well-designed survey on 1,000 Taiwanese adults [63], it is possible to calculate that the point RERI_SM/DR/BQ_ estimate for leukoplakia is higher than zero. Therefore, awareness campaigns must consider SM, DR, BQ usages as a unique unhealthy lifestyle and should focus on the behaviour of individuals who are prone to be multi-exposed and are unlikely to quit only one or two of these unhealthy behaviours [65]–[66].

In conclusion, the present meta-analysis of observational studies from South-East Asia shows that the smoking-drinking-betel quid chewing interaction has the power to increase the oral cancer risk by twenty-three to thirty-four times and such an interaction is responsible for more than two thirds (i.e., >40,000) oral cancer cases that occur in this area. The reported association between oral cancer and the smoking-drinking-betel quid chewing joint effect must, therefore, be seriously regarded in the design of effective oral cancer control policies in this area.

## Supporting Information

Appendix S1 Primary study selection: flow chart.
(DOCX)

Appendix S2 Oral cancer odds ratios (ORs, 95% confidence intervals in brackets), for the various exposure categories extracted or estimated from the primary studies.
(DOCX)

Appendix S3 Funnel plots with the lnORs of the primary studies in the *x*-axis and precision, that is, the inverse of the standard errors (se) of lnORs, in the *y*-axis. Symmetrical funnel plots denote that the degree of publication bias was probably minimal.
(DOCX)

Appendix S4 Between-study heterogeneity (Cochran's Q, χ^2^ test with 13 degrees of freedom), in the various exposure categories.
(DOCX)

Appendix S5 Relative weights (expressed as % of the overall weight) of the primary studies for the various exposure categories.
(DOCX)

Appendix S6 Sensitivity analysis to inclusion criteria for studies which provided relative weights >20% of the overall weight in a given exposure category.
(DOCX)

Checklist S1 PRISMA Checklist.
(DOCX)
