# Supplementary material for: The Magnitude of Tobacco Smoking-Betel Quid Chewing-Alcohol Drinking Interaction Effect on Oral Cancer in South-East Asia. A Meta-Analysis of Observational Studies
Source: PLoS One. 2013 Nov 18;8(11):e78999. doi: 10.1371/journal.pone.0078999 (PMC3832519; doi:10.1371/journal.pone.0078999)
Supplement: Appendix S1 — Primary study selection: flow chart. (DOCX) [file pone.0078999.s001.docx]

Studies provided by the databases

1,004 since 1996 SCOPUS

3,210 since 1996 GOOGLE SCHOLAR

203 since 1987 MEDLINE, through PubMed and Ovid

Papers selected for full text review

on the basis of titles and abstracts

84

Papers which did not focus on oral cancer or on exposures to smoking, drinking, betel quid chewing

47

Papers which focused on oral cancer and on

exposures to smoking, drinking, betel quid chewing

36

Cases and controls stratified for all the exposure categories were not

reported and corresponding authors did not provide them

22

Papers which reported cases and controls stratified for all the exposure categories;

papers with stratified data provided by the corresponding authors

14
