# Supplementary material for: The Magnitude of Tobacco Smoking-Betel Quid Chewing-Alcohol Drinking Interaction Effect on Oral Cancer in South-East Asia. A Meta-Analysis of Observational Studies
Source: PLoS One. 2013 Nov 18;8(11):e78999. doi: 10.1371/journal.pone.0078999 (PMC3832519; doi:10.1371/journal.pone.0078999)
Supplement: Appendix S2 — Oral cancer odds ratios (ORs, 95% confidence intervals in brackets), for the various exposure categories extracted or estimated from the primary studies. (DOCX) [file pone.0078999.s002.docx]

| First author  Year (number) | SM | DR | BQ | SM/DR | SM/BQ | DR/BQ | SM/DR/BQ |
| --- | --- | --- | --- | --- | --- | --- | --- |
|  |  |  |  |  |  |  |  |
| Chang  2011 (1) | 5.7  (3.7-8.7) | 1.7  (0.7-3.9) | 9.3  (2.8-30.7) | 10.0  (6.5-15.5) | 28.4  (16.7-48.5) | 20.4  (8.3-50.3) | 46.9  (33.2-66.4) |
| Lee  2012 (2) | 1.9  (1.8-2.0) | 2.3  (2.1-2.6) | 30.6  (27.3-34.3) | 4.1  (3.9-4.4) | 47.2  (45.4-49.1) | 76.7  (67.5-87.2) | 80.4  (78.0-82.9) |
| Lin  2011 (3) | 5.1  (3.2-8.3) | 1.3  (0.5-3.7) | 12.2  (3.5-40.3) | 9.9  (6.1-16.1) | 26.6  (14.5-48.6) | 21.8  (8.0-59.4) | 46.9  (31.8-69.0) |
| Lohe  2010 (4) | 2.1  (0.7-6.7) | 3.5  (0.04-331.2) | 4.9  (2.5-9.6) | 3.5  (0.04-331.2) | 4.4  (1.5-12.8) | 0.4  (0.03-7.5) | 14.0  (0.6-304.6) |
| Tsai  2009 (5) | 3.3  (2.3-4.6) | 1.0  (0.5-2.0) | 92.1  (62.9-135.0) | 2.0  (1.4-2.9) | 48.8  (37.5-63.5) | 33.6  (22.1-50.9) | 48.6  (37.8-62.6) |
| Yen,  2008 (6) | 4.7  (2.7-7.9) | 0.9  (0.3-3.1) | 11.0  (3.2-37.3) | 9.0  (5.4-15.2) | 21.8  (11.1-42.9) | 16.1  (5.3-48.4) | 39.7  (26.0-60.4) |
| Subapriya  2007 (7) | 11.4  (10.4-12.6) | 35.6  (20.1-63.0) | 5.4  (5.2-5.6) | 9.5  (7.0-12.9) | 4.5  (3.4-6.0) | 7.3  (5.5-9.6) | 14.6  (12.9-16.5) |
| Yang  2007 (8) | 5.0  (2.5-9.6) | 0.5  (0.1-3.4) | 12.8  (2.8-57.9) | 10.7  (5.7-20.1) | 28.4  (12.7-63.8) | 14.0  (3.1-64.6) | 49.8  (29.4-84.4) |
| Ko  1995 (9) | 6.8  (3.5-13.2) | 3.9  (1.6-9.4) | 36.8  (9.8-138.5) | 7.9  (4.4-14.1) | 32.7  (16.2-65.8) | 36.8  (9.8-138.5) | 41.8  (24.0-72.9) |
| Muwonge  2008 (10) | 1.2  (1.0-1.3) | 1.2  (0.7-2.2) | 4.4  (3.8-5.0) | 2.6  (2.3-2.8) | 5.3  (4.7-6.0) | 5.7  (5.0-6.5) | 4.6  (4.2-5.0) |
| Znaor  2003 (11) | 3.0  (2.9-3.0) | 2.6  (2.4-2.8) | 10.5  (10.3-10.7) | 7.7  (7.6-7.8) | 14.9  (14.6-15.2) | 1.8  (1.5-2.1) | 29.6  (29.0-30.1) |
| Sankaranarayanan  1989 (12) | 3.7  (2.6-5.0) | 0.7  (0.1-5.8) | 9.0  (7.3-11.0) | 17.6  (10.5-29.3) | 17.2  (13.9-21.2) | 21.4  (16.6-27.6) | 24.3  (17.5-33.8) |
| Sankaranarayanan  1990 (13) | 4.1  (1.8-9.1) | 2.6  (0.8-8.4) | 14.1  (7.5-26.6) | 8.5  (2.3-31.8) | 21.0  (10.7-41.2) | 20.1  (9.1-44.0) | 31.4  (13.1-74.9) |
| Rao  1994 (14) | 2.1  (2.0-2.2) | 1.5  (1.2-2.0) | 4.6  (4.4-4.7) | 2.7  (2.5-2.8) | 4.1  (4.0-4.3) | 3.8  (3.5-4.0) | 9.7  (8.8-10.8) |
