# Supplementary material for: The Magnitude of Tobacco Smoking-Betel Quid Chewing-Alcohol Drinking Interaction Effect on Oral Cancer in South-East Asia. A Meta-Analysis of Observational Studies
Source: PLoS One. 2013 Nov 18;8(11):e78999. doi: 10.1371/journal.pone.0078999 (PMC3832519; doi:10.1371/journal.pone.0078999)
Supplement: Appendix S4 — Between-study heterogeneity (Cochran's Q, χ2 test with 13 degrees of freedom), in the various exposure categories. (DOCX) [file pone.0078999.s004.docx]

| Smoking | Drinking | Chewing | χ^2^ test |
| --- | --- | --- | --- |
|  |  |  |  |
| YES | NO | NO | 193.815 ^a^ |
| NO | YES | NO | 0.008 |
| NO | NO | YES | 0.057 |
| YES | YES | NO | 0.003 |
| YES | NO | YES | 0.046 |
| NO | YES | YES | 0.008 |
| YES | YES | YES | 0.024 |

^a^ p<0.001
