# Supplementary material for: The Magnitude of Tobacco Smoking-Betel Quid Chewing-Alcohol Drinking Interaction Effect on Oral Cancer in South-East Asia. A Meta-Analysis of Observational Studies
Source: PLoS One. 2013 Nov 18;8(11):e78999. doi: 10.1371/journal.pone.0078999 (PMC3832519; doi:10.1371/journal.pone.0078999)
Supplement: Appendix S5 — Relative weights (expressed as % of the overall weight) of the primary studies for the various exposure categories. (DOCX) [file pone.0078999.s005.docx]

| Study number | Exposure category | | | | | | |
| --- | --- | --- | --- | --- | --- | --- | --- |
|  | SM | DR | BQ ^a^ | SM/DR | SM/BQ ^a^ | DR/BQ | SM/DR/BQ |
|  |  |  |  |  |  |  |  |
| 1 | 8.4% | 13.1% | 1.8% | 11.8% | 8.7% | 8.4% | 15.1% |
| 2 | 8.3% | 21.7% ^b^ | 6.0% | 11.1% | 16.0% | 13.9% | 15.2% |
| 3 | 8.3% | 8.0% | 1.8% | 9.4% | 6.8% | 7.0% | 12.0% |
| 4 | 4.4% | 0.5% | 1.0% | 0.1% | 0.6% | 0.6% | 0.1% |
| 5 | 6.7% | 3.3% | 1.8% | 1.6% | 2.4% | 4.3% | 1.8% |
| 6 | 7.0% | 1.7% | 0.5% | 2.2% | 1.4% | 1.5% | 2.6% |
| 7 | 8.0% | 4.2% | 17.7% | 1.9% | 2.2% | 6.4% | 3.8% |
| 8 | 6.3% | 0.6% | 0.3% | 1.5% | 1.0% | 0.8% | 1.7% |
| 9 | 5.5% | 2.7% | 0.5% | 1.0% | 0.9% | 1.3% | 0.8% |
| 10 | 7.9% | 4.0% | 4.8% | 5.6% | 5.3% | 13.3% | 5.0% |
| 11 | 8.6% | 28.9% ^b^ | 36.0% ^b^ | 40.4% ^b^ | 31.0% ^b^ | 9.1% | 25.8% ^b^ |
| 12 | 6.8% | 1.1% | 3.3% | 1.1% | 3.0% | 6.9% | 1.4% |
| 13 | 5.6% | 1.7% | 1.7% | 0.3% | 1.4% | 2.9% | 0.6% |
| 14 | 8.4% | 8.5% | 20.6% ^b^ | 11.9% | 16.4% | 23.7% ^b^ | 4.5% |

^a^ the sum of the study weights did not equal to 100% because of correction for publication bias

^b^ sensitivity analysis required
