# Supplementary material for: The Magnitude of Tobacco Smoking-Betel Quid Chewing-Alcohol Drinking Interaction Effect on Oral Cancer in South-East Asia. A Meta-Analysis of Observational Studies
Source: PLoS One. 2013 Nov 18;8(11):e78999. doi: 10.1371/journal.pone.0078999 (PMC3832519; doi:10.1371/journal.pone.0078999)
Supplement: Appendix S6 — Sensitivity analysis to inclusion criteria for studies which provided relative weights >20% of the overall weight in a given exposure category. (DOCX) [file pone.0078999.s006.docx]

| Study number | Exposure category | Pooled OR (95% CI) | Overall pooled OR (95% CI) |
| --- | --- | --- | --- |
|  |  |  |  |
| 2 | DR | 2.16 (1.53 – 3.05) | 2.20 (1.62 – 2.98) |
| 11 | DR | 2.06 (1.43 – 2.96) | 2.20 (1.62 – 2.98) |
| 11 | BQ | 6.73 (5.49 – 8.25) | 7.90 (6.71 – 9.30) |
| 14 | BQ | 9.10 (7.59 – 10.93) | 7.90 (6.71 – 9.30) |
| 11 | SM/DR | 5.48 (4.51 – 6.66) | 6.29 (5.41 – 7.32) |
| 11 | SM/BQ | 15.76 (12.99 – 19.14) | 16.01 (13.67 – 18.75) |
| 14 | DR/BQ | 14.35 (10.61 – 19.42) | 10.44 (8.02 – 13.60) |
| 11 | SM/DR/BQ | 44.58 (38.15 – 52.06) | 40.09 (35.06 – 45.83) |

The differences between the pORs estimated without the primary studies from the overall pORs were not statistically significantly different at 95% level.
