# Supplementary material for: The Magnitude of Tobacco Smoking-Betel Quid Chewing-Alcohol Drinking Interaction Effect on Oral Cancer in South-East Asia. A Meta-Analysis of Observational Studies
Source: PLoS One. 2013 Nov 18;8(11):e78999. doi: 10.1371/journal.pone.0078999 (PMC3832519; doi:10.1371/journal.pone.0078999)
Supplement: Supplementary file 7 [file pone.0078999.s003.docx]

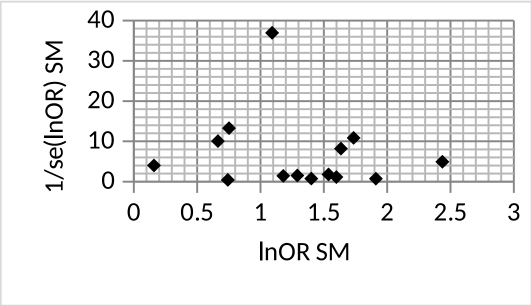
1/se(lnOR) SM
40
30
20
10
0
0 0.5 1 1.5 2 2.5 3
lnOR SM

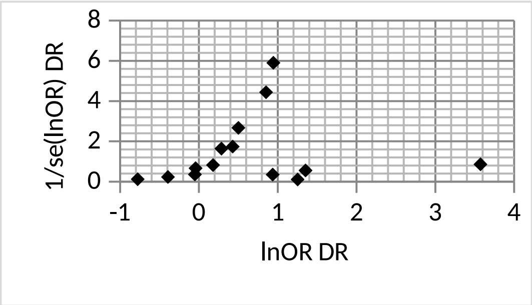
1/se(lnOR) DR
8
6
4
2
0
-1 0 1 2 3 4
lnOR DR

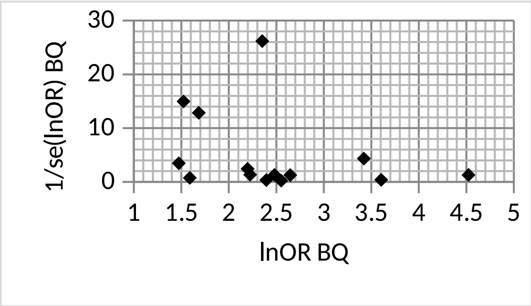
1/se(lnOR) BQ
30
20
10
0
1 1.5 2 2.5 3 3.5 4 4.5 5
lnOR BQ

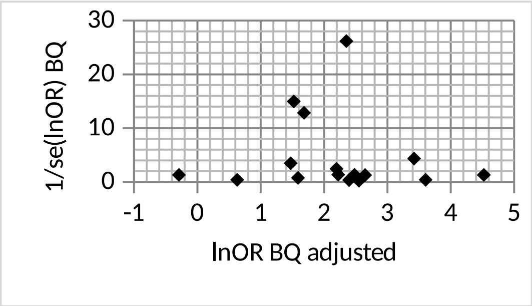
1/se(lnOR) BQ
30
20
10
0
-1 0 1 2 3 4 5
lnOR BQ adjusted

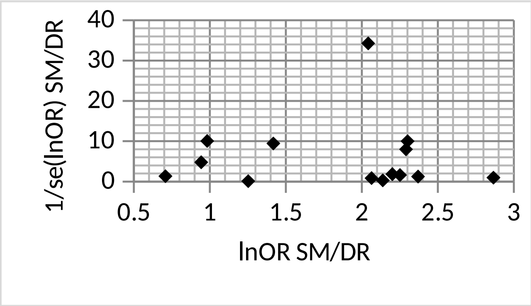
1/se(lnOR) SM/DR
40
30
20
10
0
0.5 1 1.5 2 2.5 3
lnOR SM/DR

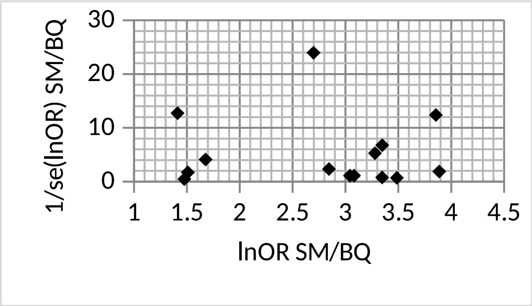
1/se(lnOR) SM/BQ
30
20
10
0
1 1.5 2 2.5 3 3.5 4 4.5
lnOR SM/BQ

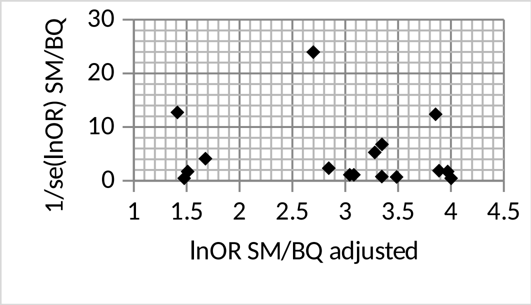
1/se(lnOR) SM/BQ
30
20
10
0
1 1.5 2 2.5 3 3.5 4 4.5
lnOR SM/BQ adjusted

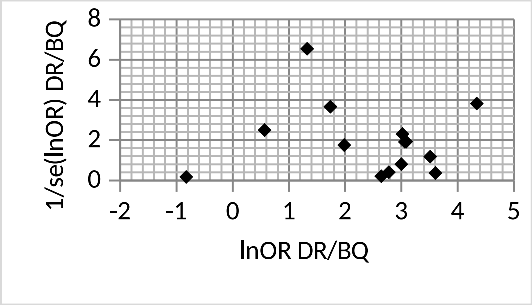
1/se(lnOR) DR/BQ
8
6
4
2
0
-2 -1 0 1 2 3 4 5
lnOR DR/BQ

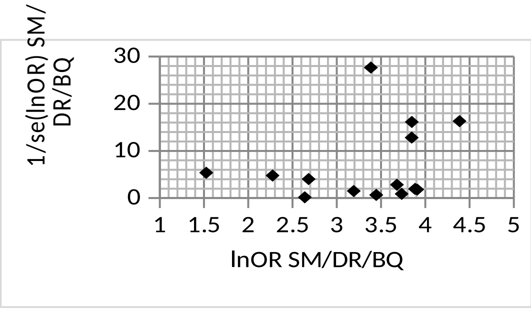
1/se(lnOR) SM/DR/BQ
30
20
10
0
1 1.5 2 2.5 3 3.5 4 4.5 5
lnOR SM/DR/BQ

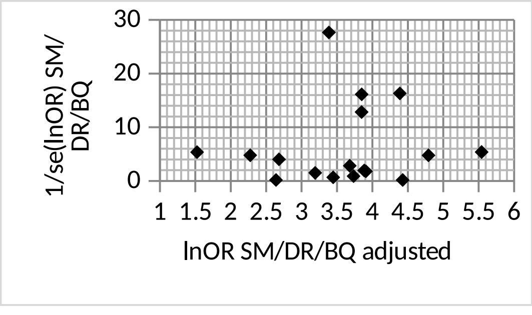
1/se(lnOR) SM/DR/BQ
30
20
10
0
1 1.5 2 2.5 3 3.5 4 4.5 5 5.5 6
lnOR SM/DR/BQ adjusted
